# Validation of the hospital resources assessment scale for the preservation of urinary continence in the elderly

**DOI:** 10.1590/0034-7167-2022-0805

**Published:** 2023-11-27

**Authors:** Roberta Pereira Góes, Larissa Chaves Pedreira, João Paulo de Almeida Tavares, Simone da Silva Oliveira, Elaine de Oliveira Souza, Fernanda Cajuhy dos Santos

**Affiliations:** I Universidade Federal da Bahia. Salvador, Bahia, Brazil; II Universidade de Aveiro. Aveiro, Portugal; III Universidade do Estado da Bahia. Salvador, Bahia, Brazil; IV Empresa Brazileira de Servicos Hospitalares, Hospital Universitário Professor Edgard Santos. Salvador, Bahia, Brazil

**Keywords:** Urinary Incontinence, Aged, Hospitalization, Nursing, Validation Study, Incontinencia Urinaria, Anciano, Hospitalización, Enfermería, Estudio de Validación, Incontinência Urinária, Idoso, Hospitalização, Enfermagem, Estudos de Validação.

## Abstract

**Objectives:**

to validate the internal structure of the Hospital Resources Assessment Scale for the Preservation of Urinary Continence in the Elderly.

**Methods:**

validation study of the internal structure of a scale constructed based on the Donabedian conceptual model and an integrative review, with prior content validation. The scale was applied to the target population, and 124 nurses responded to the questionnaire. Exploratory Factor Analysis was performed using the FACTOR software, employing multiple techniques.

**Results:**

a factorial model with 11 items organized into two dimensions (support for human resources and material resources) was obtained. The “physical structure” dimension was removed from the initial model and adopted as a complementary checklist to the instrument, as it was not possible to obtain a factorable model with this dimension.

**Conclusions:**

we provide a valid scale that can measure indicators, identifying weaknesses and/or strengths related to hospital resources for the preservation of urinary continence in the elderly.

## RESUMO

**Objetivos:**

validar a estrutura interna da escala de avaliação de recursos hospitalares para preservação da continência urinária de idosos.

**Métodos:**

estudo de validação da estrutura interna de escala construída com base no modelo conceitual da tríade donabediana e em revisão integrativa, cuja validação de conteúdo ocorreu em etapa anterior. Nesta proposta, a escala foi aplicada à população-alvo e 124 enfermeiras responderam ao formulário contendo a escala testada. Foi realizada Análise Fatorial Exploratória no *software* FACTOR, utilizando múltiplas técnicas.

**Resultados:**

obteve-se um modelo fatorial com 11 itens organizados em duas dimensões (apoio aos recursos humanos e recursos materiais). A dimensão “estrutura física” foi retirada do modelo inicial e adotada como um *checklist* complementar ao instrumento, pois não foi possível obter um modelo fatorável com essa dimensão.

**Conclusões:**

disponibilizamos uma escala válida que pode mensurar indicadores, identificando fragilidades e/ou potencialidades relacionadas aos recursos hospitalares para preservação da continência urinária de pessoas idosas.

## RESUMEN

**Objetivos:**

validar la estructura interna de la Escala de Evaluación de Recursos Hospitalarios para la Preservación de la Continencia Urinaria en los Ancianos.

**Métodos:**

estudio de validación de la estructura interna de una escala construida basada en el modelo conceptual de Donabedian y una revisión integrativa, con validación previa del contenido. La escala se aplicó a la población objetivo y 124 enfermeros respondieron al cuestionario. Se realizó un análisis factorial exploratorio utilizando el software FACTOR, empleando múltiples técnicas.

**Resultados:**

se obtuvo un modelo factorial con 11 ítems organizados en dos dimensiones (apoyo a los recursos humanos y recursos materiales). La dimensión “estructura física” se eliminó del modelo inicial y se adoptó como un checklist complementario al instrumento, ya que no fue posible obtener un modelo factorizable con esta dimensión.

**Conclusiones:**

proporcionamos una escala válida que puede medir indicadores, identificando debilidades y/o fortalezas relacionadas con los recursos hospitalarios para la preservación de la continencia urinaria en los ancianos.

## INTRODUCTION

Urinary continence is a condition that relies on multiple factors, including the integrity of the lower urinary tract and its neurological control, cognitive function, mobility, manual dexterity, and motivation. Moreover, clinical comorbidities and medication use can directly or indirectly affect urinary continence^(1)^.

Aging is associated with various changes that make older individuals more susceptible to partial or total urinary incontinence. These changes encompass increased collagen fibers in the bladder, leading to reduced elasticity; alterations in bladder pressure receptors, resulting in the development of overactive bladder contractions; loss of muscle density in the urethra, making it more fibrous and less flexible, potentially causing sphincter failure; weakening of the perineum in older women due to hormonal deficiencies (hypoestrogenism)^(2)^; and benign prostatic hyperplasia or prostate adenoma in older men^(3)^.

Therefore, urinary incontinence (UI) is considered a multifactorial geriatric syndrome, and alongside age-related intrinsic factors, modifiable and preventable factors during hospitalization can contribute to its onset or exacerbation^(4-5)^. National epidemiological data reveals that UI affects approximately 20% of the elderly population living in the community, 50% of those in long-term care facilities, and approximately 30-60% of hospitalized individuals^(6)^. It is an underreported problem that is increasing due to the aging population phenomenon.

Hence, the management of the risks associated with this condition, aiming at its prevention and/or reduction of impacts in the hospital setting, remains an area of care that is often overlooked. Studies indicate that hospital structural factors, such as the environment and the care process itself, contribute to episodes of UI, be it temporary or persistent. Notable factors include limited encouragement of independent bathroom use, excessive bed rest restrictions, inadequate privacy, insufficient signage and guidance to bathroom locations, and indiscriminate use of urinary control devices like diapers and indwelling catheters^(7-9)^.

It is therefore crucial to consider the impact of UI as a condition that can have significant consequences for the healthcare system, affected individuals, and their families/caregivers. These consequences primarily encompass diminished quality of life, psychological factors (depression, embarrassment, social isolation), physical problems such as incontinence-associated dermatitis and urinary tract infections, economic factors (costs of absorbent devices), and environmental impacts due to increased use of non-recyclable absorbent products like diapers^(10-12)^.

Thus, an instrument for evaluating and measuring aspects related to hospital resources (human resources, material resources, and physical structure conducive or non-conducive to preserving urinary continence in older individuals) enables the generation of measurable indicators related to the situation. It promotes interventions, identifies areas for improvement, and assesses the impact of interventions by monitoring these indicators in the short, medium, and long term.

To address this gap, the “Structural Assessment Instrument for Nursing Units to Preserve Urinary Continence in Older Adults” (IAEE-CUI) was developed based on the Donabedian conceptual model and studies analyzed through an extensive literature review^(4)^. After the initial construction, the scale underwent content validation in Brazil in 2020^(7)^ through evaluation by experts experienced in the subject. Consequently, a version consisting of 24 items with a dichotomous response pattern was obtained, distributed across three dimensions: “physical structure,” “material resources,” and “human resources.”

It is important to emphasize that the validity of an instrument refers to its ability to precisely measure what it intends to measure. To obtain validity evidence for a specific measurement scale, multiple techniques must be employed. Therefore, the validation process of an instrument is considered continuous and cumulative^(13)^. To enhance the robustness of the IAEE-CUI, it was necessary to proceed with validating its internal structure through application to the target population. This fact raises the following research question: Does the IAEE-CUI provide validity evidence for its internal structure in measuring the proposed construct?

## OBJECTIVES

To validate the internal structure of the scale used to assess hospital resources for preserving urinary continence in older adults.

## METHODS

### Ethical considerations

The study adhered to the principles, guidelines, and regulations governing research involving human subjects, in accordance with Resolutions 466/2012 and 510/2016 of the National Health Council, as well as the norms established for research conducted in virtual environments^(14)^. The project received approval from the Research Ethics Committees of the three participating institutions.

### Study period and location

This study focused on validating the internal structure of a scale that was constructed based on the Donabedian conceptual model and an integrative review. In the initial phase of the validation process, content validation was performed^(7)^. In this current study, we proceeded to evaluate the validity of the scale’s internal structure, including its items and dimensions, by applying it to the target population.

This stage was carried out in three hospitals located in Salvador, Bahia, Brazil: a public university hospital (Hospital A), a private hospital (Hospital B), and a philanthropic hospital (Hospital C). The data collection took place in these facilities between March and August 2021.

The target population for the study consisted of registered nurses working in wards that provide care for older adults. Hospital A had 12 wards of this nature, employing 99 registered nurses. Hospital B had four wards with 36 registered nurses, while Hospital C had 11 wards with 84 registered nurses. Therefore, the study population comprised a total of 219 nurses working across the selected 27 wards.

When determining the sample size, we followed the generally accepted rule for validating the internal structure of an instrument using Exploratory Factor Analysis (EFA). According to this rule, the sample should consist of at least five times the number of respondents as there are items in the scale^(15)^. Therefore, for this study, our goal was to achieve a sample size of at least 120 nurses, considering the 24 items of the IAEE-CUI scale.

The inclusion criteria were as follows: having a minimum of three months of experience as a registered nurse in the respective unit, agreeing to participate in the research, and not being on vacation or on leave during the data collection period. The final sample included 124 nurses who responded to the questionnaire (64 from Hospital A, 20 from Hospital B, and 40 from Hospital C).

The study protocol forms part of a doctoral thesis entitled “ *Construção e validação da escala de avaliação de recursos hospitalares para preservação da continência urinária de idosos* ^(16)^,” which was presented to the Graduate Program in Nursing and Health at the *Universidade Federal da Bahia*, Brazil. Data collection for this phase of the study was conducted remotely using the Google Forms platform to administer two self-administered questionnaire instruments. The instruments were accompanied by the Informed Consent Form (ICF), which was sent via a link. After reading the ICF and providing consent, the nurses gained access to the questionnaire, which included a participant characterization form with continuous and categorical variables, as well as the 24 items of the IAEE-CUI scale (dichotomous categorical variables).

### Analysis of Results and Statistics

The collected data were transferred and organized in an electronic database and analyzed using descriptive statistics. Mean and standard deviation were calculated for continuous variables, while relative frequency and percentage were calculated for categorical variables, using Microsoft Office Excel (2013) software.

**Figure 1:**
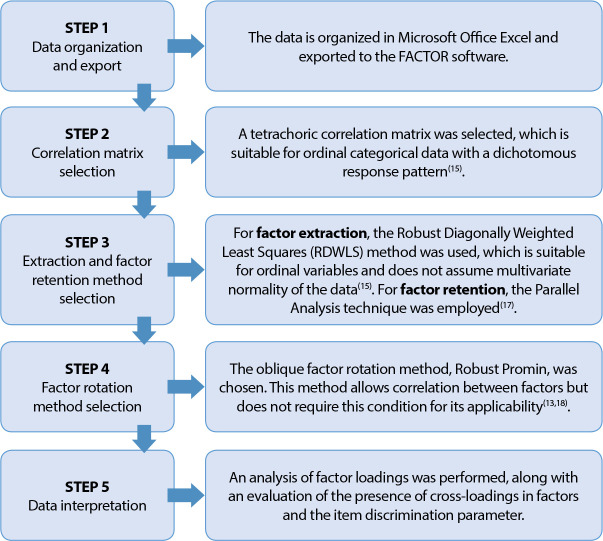
Association of the five necessary steps for conducting an Exploratory Factor Analysis and the selected techniques for validating the internal structure of the instrument

The validity of the internal structure of the IAEE-CUI scale was assessed through Exploratory Factor Analysis (EFA) conducted using the FACTOR software (2006-2021), employing various techniques. It is important to highlight that the necessary techniques were selected to fulfill the five essential steps of EFA and their assumptions^(15)^. These steps, detailed and justified in Figure 1, were planned and selected based on the IAEE-CUI instrument model and the nature of its items/variables.

As an initial step for conducting an Exploratory Factor Analysis (EFA), the suitability of the data matrix for factorization was assessed. The Kaiser-Meyer-Olkin (KMO) criterion and Bartlett’s Test of Sphericity were utilized for this purpose^(15)^. Subsequently, during the analysis of the factor matrix, the following criteria were applied to guide the decision-making process regarding the retention or exclusion of items from the instrument: (1) evaluation of factor loading saturation, with items having a factor loading ≥ 0.30 being retained; (2) analysis of the presence or absence of items with cross-loadings on factors; (3) consideration of the practical relevance and conceptual meaning of the evaluated item in relation to the factor/dimension to which it belongs and the construct being measured by the scale^(15)^.

As supplementary criteria, unidimensionality indices were also assessed using the parameters of Unidimensional Congruence (UniCo), Explained Common Variance (ECV), and Mean of Absolute Residual Loadings (MIREAL)^(19)^. The item discrimination parameter was evaluated using Reckase’s parameterization, which indicates the item’s level of discrimination for a specific factor^(20)^. Schwarz’s Bayesian Information Criterion (BIC) measure was employed for model comparison^(19)^.

## RESULTS

Regarding the participants’ characterization, the respondents had a mean age of 36.1 ± 5.9 years, ranging from 22 to 54 years, with the highest mean age in Hospital A (37.6 ± 5.8). The mean duration of nursing experience was 9.4 ± 5.8 years, ranging from 6 months to 30 years, with the highest mean in Hospital A (11.8 ± 5.7). In terms of the duration of employment in the unit, the mean was 3.5 ± 3.4 years, ranging from 4 months to 18 years, with the highest mean in Hospital C (4.7 ± 4.7). As for gender, 87.5% of the sample identified as female.

Regarding the time dedicated to managing urinary continence during the work shift, the mean was 1.3 ± 2.3 hours, ranging from 0 to 12 hours, with the highest mean in Hospital C (2.3 ± 3.7), considering a 12-hour work shift as a reference.

The majority of participants reported having a specialization in the nursing field (87.1%), with Hospital B having the highest percentage (100%). However, there was a general trend of low participation in training or capacity-building related to continence care in the hospital setting (19.3%), with the lowest rate in Hospital A (9.4%), followed by Hospital B with 20% and Hospital C with 35%.

Only 19.3% of the participants reported being familiar with any protocols or guidelines related to urinary incontinence (UI), with the lowest rate in Hospital A (4.7%). When it comes to evaluating the patient’s urinary continence pattern upon admission, 21.8% of the sample reported not performing this assessment. Additionally, 34.7% of the nurses reported not being able to differentiate between transient and permanent UI in their clinical practice, with the highest rate in Hospital A (40.6%), followed by Hospital B with 30% and Hospital C with 27.5%.

Regarding the validation of the instrument’s internal structure, the Exploratory Factor Analysis (EFA) conducted with data from the three dimensions together resulted in a non-factorable matrix. In other words, the tested factor structure was not suitable for obtaining the planned indicators for EFA in the FACTOR software. However, when separate EFAs were conducted for each dimension, considering their interdependence, the matrix was factorable for each of the three dimensions. Nonetheless, significant issues were identified in the second dimension/factor, “Physical Structure,” where many variables/items had factor loadings below the acceptable threshold (0.30).

Furthermore, upon analyzing the item-item correlation matrix of this specific dimension/factor, a low correlation between its variables was observed. The items assessing the physical structure were interdependent attributes that did not have sufficient covariance to form a common factor in an EFA, leading to the decision to remove this dimension.

However, considering the practical relevance of evaluating the physical structure of the unit, the evaluated construct, the theoretical framework used in constructing the instrument, and the achieved content validation after constructing these items^(7)^, it was decided to retain this dimension as a complementary checklist to the instrument. The proposed subscale in checklist format (Chart 1) was separated from the instrument, and the model, now proposed with two dimensions/factors (human resources and material resources), was re-specified and subjected to a new EFA.

**Chart 1:** Checklist for assessing the physical structure of wards for the preservation of urinary continence in older adults

Physical structure resources of the ward for preserving urinary continence in older adults:	Yes	No
Are there grab bars in the bathroom near the toilet?		
Are there grab bars or handrails along the path from the bed to the bathroom?		
Are there doors in the bathrooms of each ward?		
Are the bathroom doors sliding doors?		
Is the flooring in the bathroom and ward non-slip?		
Is the flooring in the bathroom and ward non-glare?		
Are the bathrooms in the ward always kept clean?		
Are the bathrooms in the ward always kept with a pleasant odor?		
Is the location of the bathrooms clearly marked?		
Is there sufficient space in the bathroom to maneuver a commode chair or wheelchair?		
**Action plan in the short, medium, and long term for items with a negative response:**		

The revised structure of the instrument consists of 14 items (variables), with six items in the “human resources” dimension and eight items in the “material resources” dimension. In terms of the number of factors to retain, the parallel analysis technique suggested retaining two factors, which confirms the previously defined bidimensional specification model. This model accounted for a total explained variance of 48.17% (29.62% for Factor 1 and 18.55% for Factor 2). When evaluating the indicators of unidimensionality, none of these indices indicated a unidimensional model for this matrix (UniCo: 0.744; ECV: 0.632; and MIREAL: 0.338), further supporting the evidence of the two-factor structure.

Regarding the analysis of factor loading saturation, Table 1 demonstrates that the dimension previously referred to as “human resources” (V1 to V6) had Variable 1 (an item questioning the evaluation of adequate nursing staffing for workload in the unit) with a factor loading <0.30. While adequate nursing staffing is relevant to the evaluated construct, it is already evaluated in many hospital units using nationally validated instruments. Consequently, it was decided to remove this item from the instrument after careful consideration.

**Table 1:** Factorial matrix of the two-dimensional tested model containing the factor loadings of the analyzed items, Salvador, Bahia, Brazil, 2021

Item/Variable Specification	Factor 1	Factor 2
	Human Resources	Material Resources
V1. Adequate nursing staffing.	-0.130	-0.020
V2. Criteria for diaper use.	0.686	0.032
V3. Criteria for urinary catheter use.	0.704	-0.127
V4. Nursing process with interventions aimed at minimizing or improving UI in older adults in the unit.	0.693	0.048
V5. Presence of an instrument for evaluating UI in the unit.	0.487	0.068
V6. Provision of ongoing education related to UI in the unit.	0.448	0.002
V7. Adequate availability of privacy materials such as screens and curtains.	0.009	0.737
V8. Presence of automatic beds and/or bedside commodes near the bed.	0.026	0.092
V9. Sufficient number of bedpans for the demand.	-0.100	0.590
V10. Sufficient number of urinals for the demand.	-0.086	0.532
V11. Presence of individual lighting at the bedside.	0.290	0.560
V12. Presence of lighting sensors in the unit.	0.319	0.563
V13. Sufficient availability of materials to assist with mobility to the bathroom.	-0.132	0.726
V14. Sufficient availability of commode chairs in the unit.	-0.255	0.562
Explained variance (48.17%)	29.62%	18.55%

Following the removal of V1, the dimension/factor was renamed as “support for human resources” since an analysis of the remaining variables (V2 to V6) revealed that they were more related to supporting human resources in continence care rather than the human resources themselves. Therefore, it was deemed more appropriate to label it as “Support for human resources.” The analysis and decision concerning the internal structure of the items in this dimension were concluded.

The analysis of the variables/items in the “material resources” dimension (V7 to V14) was then conducted. Among these variables, it was found that V8 had a factor loading <0.30. This item pertains to evaluating the presence of automatic beds or, in their absence, support stairs for safe bed egress.

Descriptive data revealed limited response variability even when applying the instrument in three different hospital institutions (94.35% of respondents provided a positive response). This suggests that the availability of automatic beds or support stairs for safe bed egress is widespread in these settings. Consequently, due to the near-unanimous response in the sample, there was insufficient variability for this item to be discriminative. When an item lacks discriminative ability in a measurement instrument, its exclusion may be considered.

To strengthen this decision, the Reckase parameterization criterion was employed. Based on the factor matrix of item discrimination patterns, it was determined that this variable (V8) demonstrated the least discriminative power in the entire instrument, with a value of 0.093. Hence, the decision was made to exclude it.

Furthermore, when examining the variables in the “material resources” dimension, it was observed that V12 (an item regarding the presence of lighting sensors in the unit) exhibited cross-loadings on both factors (factor loading above 0.30). Theoretically, this item belongs to the “material resources” dimension. However, considering that the attribute of “lighting” is also addressed in item 11 of this dimension and the fact that V12 showed cross-loadings on both factors, it was decided to exclude it and re-specify the model.

Consequently, at the conclusion of the interpretation of this factor matrix, variables/items 1, 8, and 12 were excluded, resulting in a re-specified model with two dimensions/factors and eleven items. The factor solution of this model demonstrated a well-defined internal structure (Table 2), with all variables exhibiting acceptable factor loadings on their respective factors without cross-loadings.

**Table 2:** Factor matrix of the adjusted model of the instrument, showing the highlighted factor loadings of the items on their respective factors/dimensions, Salvador, Bahia, Brazil, 2021

Specification of Items/Variables	Factor 1	Factor 2
	Support for Human Resources	Material Resources
V1. Criteria for diaper usage.	**0.926**	0.184
V2. Criteria for urinary catheter usage.	**1.000**	-0.003
V3. Interventions to minimize or improve urinary incontinence integrated into the nursing process.	**0.644**	0.044
V4. Assessment tool for evaluating urinary incontinence in the unit.	**0.496**	0.048
V5. Ongoing education provided regarding urinary incontinence in the unit.	**0.416**	-0.119
V6. Adequate privacy materials, such as screens and curtains.	0.072	**0.611**
V7. Sufficient number of bedpans to meet the demand.	0.119	**0.866**
V8. Adequate number of urinals to meet the demand.	0.075	**0.898**
V9. Individual lighting at the head of each bed.	0.227	**0.382**
V10. Sufficient materials to assist with mobility to the bathroom.	-0.120	**0.861**
V11. Adequate number of commode chairs available in the unit.	-0.201	**0.763**
Explained variance (60.51%)	35.50%	25.01%

Hence, the EFA demonstrated that the adjusted model with eleven items provided the most parsimonious factor solution for validating the internal structure of the scale. Additionally, when comparing the Bayesian Information Criterion (BIC) value of the previous model (BIC: 321.013) with that of the adjusted model (BIC: 249.192), a reduction in the value was observed, indicating that the adjusted model is more parsimonious.

After selecting the model to be adopted as the factorial solution, further consideration was given to determining the most appropriate name for the scale, taking into account the two dimensions and the remaining items/variables. It was determined that the instrument would be named the “Hospital Resources Evaluation Scale for the Preservation of Urinary Continence in Older Adults,” abbreviated as REHOSP-CUI-11.

**Figure 2:**
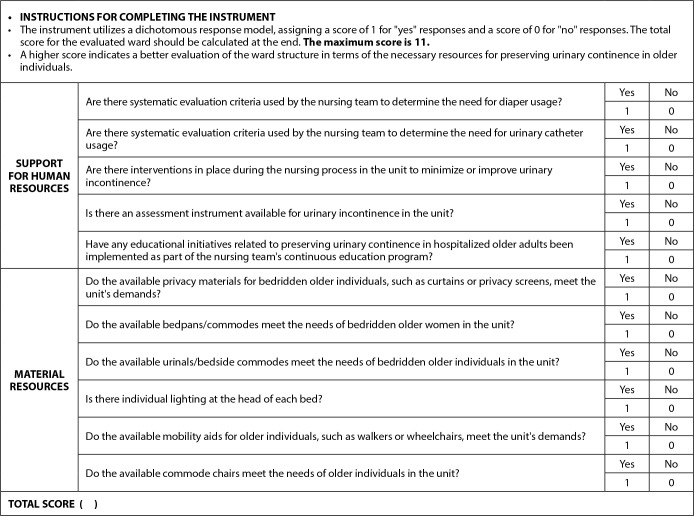
Hospital Resources Evaluation Scale for Preserving Urinary Continence in Older Adults (REHOSP-CUI-11)

The updated version presented in Figure 2 has a maximum score of 11 points and maintains a dichotomous response format (yes/no). A score of 0 is assigned to “no” responses, while a score of 1 is assigned to “yes” responses. This scoring system indicates that a higher score reflects a more suitable ward structure in terms of the necessary resources for preserving urinary continence in older individuals.

Thus, to establish the structural diagnosis of indicators in a ward related to resources for preserving urinary continence in hospitalized older adults, it is suggested to: (1) administer the REHOSP-CUI-11 instrument; (2) use the complementary checklist for assessing the physical structure (a tool that can be attached to the instrument during its application in practice); and (3) evaluate the adequacy of nursing staffing in the unit using a nationally validated instrument for this purpose.

## DISCUSSION

The sample characterization data indicated a slight but noticeable involvement of Hospital C in the management of urinary continence. There was a notable lack of participation from nurses in all participating institutions in training or education related to continence care in the hospital setting. Additionally, participants showed a knowledge deficit regarding protocols or guidelines related to urinary incontinence, and a considerable number of respondents reported not routinely assessing urinary continence patterns upon patient admission and being unable to differentiate between transient and permanent incontinence.

These results serve as an important alert to guide intervention measures aimed at the continuous education of the nursing team regarding continence care and the management of urinary incontinence risks in hospitalized older adults. This discussion and the need for training can also be extended to other members of the multidisciplinary team working in the hospital setting.

These findings are consistent with a study conducted in South Korea aimed at developing an online continuing education course for nurses on continence care and evaluating its effectiveness. This study revealed the need for the educational intervention conducted and the impact of the program on nurses’ knowledge and attitudes towards continence care. The authors suggested that to improve outcomes for populations affected by incontinence, managers, educators, researchers, and clinicians need to work collectively to develop, implement, and evaluate the effectiveness of specific continuing education programs^(21)^.

It is important to note that a lack of knowledge about urinary incontinence and its consequences in the hospital setting leads to missed opportunities for providing guidance by the care team, implementing urinary incontinence risk management, and referring patients to specialists when necessary. Therefore, when an assessment focused on urinary continence status is neglected, it can lead to avoidable harm. Evidence indicates that even if identified urinary incontinence cannot be reversed, guideline-based management strategies can result in symptom improvement in up to 70% of cases^(22)^.

Regarding the results of the internal structure validation of the instrument, it was found that eleven out of the initially proposed 24 items were validated through Exploratory Factor Analysis (EFA), with retention of two factors as indicated by the Parallel Analysis technique. The obtained factor solution showed satisfactory conditions for validation, with a significant Bartlett’s test of sphericity, an acceptable Kaiser-Meyer-Olkin (KMO) index (above 0.7), a total explained variance above 60.0%, at least three retained items in each factor, and factor loadings of all variables above 0.30^(15)^.

The decision to exclude the items was based on previously established criteria, in accordance with theoretical and methodological justifications. Regarding the item related to nursing staffing, it is presumed that the discrepancy in its factor loading in the factor matrix of the “human resources” dimension can be explained by the fact that it is the only variable related to the quantity of staff, while the others refer to attributes related to qualification and/or support instruments for human resources. Therefore, its exclusion was justified by the fact that it is not advisable to retain a single variable in another factor^(15)^, especially considering the existence of other validated instruments that measure nursing staffing in hospital ward settings^(23)^.

Regarding the item related to the availability of automatic beds or nearby stairs, there was a predominance of positive responses during the evaluation of this resource. This made the item less discriminatory, leading to the decision to exclude it. It is worth noting that this result demonstrated the potential of this material resource in different hospital settings within the study field. Automatic beds not only provide greater patient safety but also benefit the ergonomics of healthcare professionals^(22)^.

Regarding the item related to the presence of lighting sensors in the units, considering the Brazilian reality of hospital structures, although it is essential for the care unit to have adequate lighting for the compromised visual acuity of older individuals, the use of sensors is not essential^(24)^. Thus, while activation by sensors is undoubtedly preferable, it is not indispensable, considering the context of evaluation and the limitation of financial resources in the healthcare system. Therefore, this item was removed.

A set of evidence based on different criteria strengthens the fact that the instrument has a valid internal structure for measuring the assessed construct^(15,25)^. Therefore, the decision to remove or retain an item should be guided by previously established criteria, but these criteria do not dictate the decision. Rather, they guide the researcher’s interpretation, and it is plausible to consider the statistical results, the theoretical framework used for instrument construction, and the practical relevance of the item in light of the empirically found results, as was the case when certain variables were carefully excluded from the tested scale.

### Study limitations

One limitation of the study is that the REHOSP-CUI-11 scale does not yet have defined cutoff points to guide a more objective interpretation of its application. It is known that the variables measured in the two dimensions of the instrument have different impacts on the assessed construct. Therefore, future studies are needed to define these cutoff points using statistical procedures and associate the results obtained from scale application with external measures (outcome-related indicators) related to the investigated outcome, such as monitoring the incidence of urinary incontinence in older adults in the evaluated units after scale application, identifying weaknesses, and implementing actions.

Furthermore, this manuscript does not present the reliability indices of the scale measurement, as this is the subject of another study. Therefore, future studies with larger samples and application of the scale in other settings are recommended to strengthen these results.

### Contributions to the Nursing, Health, or Public Policy Field

The study provides a practical product of relevance to the nursing and healthcare field in the hospital context. The constructed scale has achieved evidence of content validity and internal structure validity of its items and dimensions, allowing for greater guidance in monitoring structure, process, and outcome indicators and managing the risk of onset/worsening of urinary incontinence in hospitalized older adults.

Furthermore, this study contributes to the description of the steps and methodological choices used, given the need to disseminate knowledge related to validation techniques of the internal structure of a measurement instrument using software and techniques that yield robust results during Exploratory Factor Analysis (EFA). In this context, the use of the FACTOR software, the Parallel Analysis technique for factor retention^(25)^, and the use of multiple techniques to obtain indicators that support the process of validity evidence and ensure more accurate decisions during the analysis process are highlighted.

## CONCLUSIONS

We have validated the internal structure of an instrument that assesses structural aspects of wards regarding the preservation of urinary continence in older adults. The empirical model had its internal structure validated in the context of general hospital wards with different profiles that serve older adults, following its application to the target population (clinical nurses) and EFA of the data, resulting in a more parsimonious two-dimensional model composed of 11 items.

We decided to exclude one of the dimensions from the instrument adopted in the initial three-factor model due to the strong interdependent nature of its variables, opting to consider the “physical structure” dimension as a complementary checklist. Therefore, we obtained the final version called the “Hospital Resources Evaluation Scale for Preserving Urinary Continence in Older Adults” (REHOSP-CUI-11), consisting of the dimensions: (1) Support for human resources and (2) Material resources.

The validated instrument allows for valuable and useful quality indicators that will measure, evaluate, and monitor the structural resources of wards in relation to the preservation of urinary continence in older adults. The results of this study, in addition to bringing visibility to the topic, alert healthcare professionals and managers to the necessary considerations in hospital architecture and human and material resource management in the face of population aging. They also emphasize the need to preserve the comfort and autonomy of older individuals, allowing for safe use of the bathroom during hospitalization to maximize the functionality of urinary continence throughout the hospitalization process.
